# Salivary Nitrate Level and Lipid Profile in Patients with Hypertension: A Cross-Sectional Study in a Saudi Sub-Population

**DOI:** 10.3390/jcm13237051

**Published:** 2024-11-22

**Authors:** Khalil Ibrahim Assiri, Ali Mosfer A. Alqahtani, Abdullah Alqarni, Hassan Ahmed Assiri, Saeed Alassiri, Samiunnisa Begum Shaik, Ali Azhar Dawasaz, Mohammad Shahul Hameed

**Affiliations:** 1 Department of Diagnostic Sciences and Oral Biology, College of Dentistry, King Khalid University, Abha 62529, Saudi Arabia; alasery@kku.edu.sa (K.I.A.); al.alqahtani@kku.edu.sa (A.M.A.A.); aawan@kku.edu.sa (A.A.); halmuawad@kku.edu.sa (H.A.A.); sadelboh@kku.edu.sa (S.A.); adwasaz@kku.edu.sa (A.A.D.); 2 Department of Oral Medicine and Radiology, Ragas Dental College & Hospital, Chennai 600119, India; samriyah1794@gmail.com

**Keywords:** hypertension, triglycerides, nitric oxide, saliva, HDL lipoproteins, LDL lipoproteins

## Abstract

**Background**: The use of salivary biomarkers offers a non-invasive approach to understanding the metabolic and inflammatory status of hypertensive patients. This study aimed to quantify the salivary nitric oxide (NO), total cholesterol, triglycerides, high-density lipoproteins (HDL), and low-density lipoproteins (LDL) levels in hypertensive individuals and healthy controls in a sub-population in Saudi Arabia. **Methods**: This cross-sectional study comprised 40 hypertensive patients (test group, 40–50 years old) and 40 age-matched healthy controls who visited the dental hospital in the College of Dentistry, King Khalid University, for dental treatment. Nitric oxide, total cholesterol, triglycerides, HDL, and LDL levels in saliva were assessed. An independent sample t-test was used to compare the results between the hypertensive and control groups. **Results**: The mean triglyceride and cholesterol levels in the test group were significantly higher (*p* < 0.05) than those in the control group. Alternatively, the NO level in the test group was significantly (*p* = 0.014) lower than that in the controls. The triglyceride level was significantly correlated with age in the test group (*p* = 0.04). **Conclusions**: This study demonstrated significant differences in the nitrate levels and lipid profiles between hypertensive patients and healthy individuals in a sub-population in Saudi Arabia. The findings indicate that saliva can be used as a non-invasive diagnostic tool for assessing nitrate levels and the lipid profile. However, additional studies with larger sample sizes and more precise testing parameters are required to validate the findings.

## 1. Introduction

Hypertension or high blood pressure is a prevalent cardiovascular condition associated with increased morbidity and mortality. A recent study, which measured the prevalence of hypertension and progress in 200 countries and territories, reported that the number of people affected by this condition had doubled from 1990 (331 million women and 317 million men) to 2019 (626 million women and 652 million men) [1].

Hypertension is associated with changes in lipid metabolism, leading to abnormal levels of serum lipids and lipoproteins. The increase in cholesterol and low-density lipoproteins (LDL) in the blood is associated with hypertension and its risk factors, such as atherosclerosis. Although cholesterol is required for numerous vital functions in the body, it is known to be involved in various pathological conditions, such as obesity, diabetes, kidney disorders, and hypertension [2]. According to a recent study, patients with hypertension are more likely to have dyslipidemia compared to healthy subjects. Hence, measurements of blood pressure and lipid profile levels, including serum total cholesterol, triglyceride, LDL, and high-density lipoproteins (HDL), at regular intervals are vital [3].

Nitric oxide (NO), a key vasorelaxing agent produced by the vascular endothelium, controls and regulates blood pressure by influencing the vascular tone. It is oxidized to stable and inert metabolites, nitrate and nitrite ions, under aerobic conditions; however, they are physiologically recycled in blood and tissue to form NO, forming the nitrate-nitrite-NO pathway [4]. Nitrates from dietary or endogenous sources are initially reduced to nitrite via the action of commensal bacteria in the oral cavity [5] and gastrointestinal tract [4]. The loss of NO production may lead to increased blood pressure, initiating the development of hypertension [6]. Nayak et al. (2016) reported significantly lower plasma NO levels in patients with hypertension than in healthy controls [7].

Local and systemic infections, including oral infections (such as periodontitis), can significantly alter the plasma concentration of cytokines, resulting in hyperlipidemia [8]. Studies have reported significant differences in lipid profile values between high-risk hypertensive patients and healthy subjects in both serum and saliva [8,9]. Furthermore, correlations between serum and salivary cholesterol levels have been reported in both healthy [10] and hypertensive individuals [11]. Thus, evaluating the salivary cholesterol level may help identify individuals with high serum cholesterol levels [10].

Whole saliva is an important physiologic fluid containing a mixture of substances, including blood and serum products [12]. The concentration of nitrate in saliva increases almost 10-fold compared to that in plasma due to active uptake by the salivary glands; this concentration is further increased following the consumption of nitrate-rich food, such as green leafy vegetables and beetroot [5,13]. Furthermore, the salivary nitrate concentration is influenced by several factors, such as the salivary flow rate [14], oral microbiome [5], and fluctuations during the day and night [15].

Salivary biomarkers can enhance our understanding of the disease mechanism and help personalize interventions. One study reported positive correlations between salivary and serum nitric oxide levels among patients with periodontitis and healthy individuals, indicating that the results of a salivary non-invasive examination significantly correlated with those of the serum analysis [16].

Studies on salivary nitrate levels and lipid profiles in hypertensive patients as non-invasive biomarkers are limited. Therefore, this study aimed to quantify the salivary NO, total cholesterol, triglycerides, HDL, and LDL levels in hypertensive individuals and healthy controls in a sub-population in Saudi Arabia. The use of salivary biomarkers offers a non-invasive approach to understanding the metabolic and inflammatory status of hypertensive patients. Understanding the interplay between salivary NO and lipid profiles in hypertensive patients and controls can help in early detection, risk determination, and the implementation of management strategies. By exploring these novel biomarkers, we aim to contribute to better dental management in hypertensive patients and follow-up through non-invasive procedures.

## 2. Materials and Methods

### 2.1. Study Design

This study employed a cross-sectional design involving diagnosed hypertensive patients and age-matched healthy controls who visited the dental hospital in the College of Dentistry, King Khalid University, for dental treatment. Informed consent for participation in the study was obtained from the patients. The study was conducted according to the guidelines of the Declaration of Helsinki, and ethical approval for the study was obtained from the scientific research committee at the College of Dentistry, King Khalid University (approval number: IRB/KKUCOD/ETH/2024-25/004).

### 2.2. Participants

Eighty participants (test group, n = 40; control group, n = 40) aged 40–50 years were included in the study (Table 1). The test group comprised patients diagnosed with essential hypertension who did not use nitrates or statins. Those with severe comorbidities affecting salivary function, those who were diagnosed with other systemic diseases, and smokers were excluded. The participants in the two groups were further subdivided based on age: subgroup 1, 40–43 years; subgroup 2, 44–46 years; and subgroup 3, 47–50 years.

### 2.3. Salivary Sample Collection

All patients included in the study underwent oral prophylaxis at the dental hospital before saliva collection. Eight-hour fasting salivary samples were collected between 8 and 10 a.m. to avoid the effects of circadian rhythm. Patients were asked to sit in an upright position, and 5 mL of unstimulated whole saliva was collected in a graduated test tube using the spitting method. The salivary samples were centrifuged at 1800 rpm for 20 min to separate the supernatants, which were transferred to sealed and labeled containers (control and test groups). The samples were transported to the laboratory in a Styrofoam box containing ice for storage at −80 °C until further use. All the samples were thawed to room temperature prior to analyses of the salivary parameters.

### 2.4. Salivary Lipid Profile Analysis

The total cholesterol, triglycerides, HDL, and LDL levels were assessed.

#### 2.4.1. Triglycerides

The triglyceride levels were estimated using a kit manufactured by Ensure Diagnostics (catalog number AAG18-2402; Ensure Biotech Pvt. Ltd., Tamil Nadu, India). Clean, dry test tubes were labeled as blank (B), standard (S), and test (T), and 1 mL of triglyceride reagent was added to the tubes with the help of a pipette. Subsequently, 10 µL of distilled water was added to tube B, 10 µL of standard (provided by the manufacturer) was added to tube S (positive control), and 10 µL of saliva sample was added to tube T. The solutions in the tubes were mixed well and incubated at 37 °C for 10 min. The optical density (OD) values of the three tubes were measured at 546 nm, and the amounts of triglycerides were calculated as follows:

TGL = A (T)/A (S) × concentration of the standard (200 mg/dL),

where A stands for the absorbance.

#### 2.4.2. Cholesterol

A similar method was used to measure the cholesterol levels utilizing the kit from Ensure Diagnostics (AA05-2402; Ensure Biotech Pvt. Ltd., Tamil Nadu, India), according to the manufacturer’s instructions. One ml of cholesterol reagent was added to clean dry test tubes labeled as B, S, and T, followed by the addition of 10 µL of distilled water to tube B, 10 µL of standard (provided by the manufacturer) to tube S (positive control), and 10 µL of saliva sample to tube T. The solutions were mixed well and incubated at 37 °C for 10 min. The OD values were measured at 505 nm, and calculations were made using the following formula:

Cholesterol concentration (mg/dL) = A (T)/A (S) × concentration of the standard (200 mg/dL)

#### 2.4.3. HDL Levels

The HDL levels were estimated using a kit manufactured by Ensure Diagnostics (catalog number AA45-2403; Ensure Biotech Pvt. Ltd., Tamil Nadu, India). One ml of HDL reagent was added to clean dry test tubes labeled as B, S, and T, followed by the addition of 10 µL of distilled water to tube B, 10 µL of standard (provided by the manufacturer) to tube S (positive control), and 10 µL of saliva sample to tube T. The solutions were mixed well and incubated at 37 °C for 10 min. The OD values were measured at 620 nm, and calculations were made using the following formula:

HDL (mg/dL) = A (T)/A (S) × concentration of the standard (40 mg/dL)

#### 2.4.4. LDL Levels

The LDL levels were estimated using a kit manufactured by Praiksha Biotech (catalog number P-LDL(D)-3228; Hyderabad, India). LDL-C R1 reagent (600 µL) from the kit was added to clean dry test tubes labeled as B, calibrator (C), and T, followed by the addition of 8 µL of distilled water to tube B, 8 µL to calibrator to tube C, and 8 µL of saliva sample to tube T. The solutions were mixed well and incubated at 37 °C for 5 min. Subsequently, 200 µL of LDL-C R2 reagent was added to all the test tubes. The OD values of the tubes were measured at 546 nm, and calculations were made using the following formula:

LDL (mg/dL) = A (T)/A (C) × concentration of the calibrator (110 mg/dL)

#### 2.4.5. Nitric Oxide

The salivary NO levels were estimated as described previously [17]. The levels were assessed as the nitrate concentration using a commercially available colorimetric assay kit (Nitric Oxide Total Detection Kit #89141-754, Enzo Life Sciences, Inc., Farmingdale, NY, USA), in accordance with the manufacturer’s instructions. The kit is based on the enzymatic conversion of nitrate to nitrite by the enzyme nitrate reductase, followed by the Griess reaction to form a colored azo dye product. Quantification was performed by measuring the absorption at 550 nm [18]

### 2.5. Statistical Analysis

The results were evaluated using Statistical Package for the Social Sciences (SPSS, Version 21; Chicago, IL, USA). Descriptive statistics are presented as mean ± the standard deviation. The independent sample *t*-test was used to compare the test variables between the test and control groups. One-way analysis of variance (ANOVA) and the least significant difference (LSD) post hoc tests were conducted to compare the variables between and among the subgroups based on age. A *p*-value of <0.05 was considered significant.

## 3. Results

Forty hypertensive patients (test group; 20 males and 20 females; age, 40–50) and 40 age-matched healthy individuals (control group; 20 males and 20 females) were included in the study. Table 1 shows the descriptive statistics of the salivary variables in the two groups.

The mean triglyceride level in the test group was 79.4 ± 20.71 mg/dL (range, 55–180 mg/dL), while that in the control was 68.85 ± 12.45 mg/dL (range, 50–95 mg/dL). Likewise, the mean cholesterol level in the test group (55.67 ± 7.14 mg/dL) was higher than that in the control group (52.20 ± 7.19 mg/dL). The HDL and LDL levels were similar in both groups. However, the mean NO level in the test group (127.4 ± 4.46 µmol/L) was lower than that in the controls (156.27 ± 5.69 µmol/L).

As shown in Table 2, the *t*-test revealed significant differences in triglyceride (*p* = 0.007), cholesterol (*p* = 0.033), and NO (*p* = 0.014) levels between the two groups.

The intervariable Pearson’s test showed a significant correlation between the triglyceride level and age in the test group (*p* = 0.04; Table 3).

Furthermore, the ANOVA and LSD post hoc analyses revealed a significant difference in triglyceride levels between subgroups (*p* = 0.034), especially subgroups 1 and 3 (*p* = 0.015) in the test group (Table 4). However, no significant associations were observed among the variables in the control group. Similarly, no significant difference was observed between males and females in any of the variables assessed.

## 4. Discussion

This study quantified and compared the salivary NO levels and lipid profiles (total cholesterol, triglycerides, HDL, and LDL) between hypertensive individuals and healthy controls.

Hypertension is a chronic cardiovascular condition that poses a major public health challenge worldwide. A recent systematic review and meta-analysis reported a high prevalence (22.7%) of hypertension, with low awareness, treatment, and control rates among Saudi patients [19]; however, these values were lower than those reported in other neighboring and Western countries. Underlying factors, such as aging, obesity, dyslipidemia, glucose intolerance, and diabetes, have been associated with this condition. Dyslipidemia is assessed by analyzing the lipid profile in the serum. NO plays a vital role in vascular control, and the predictive value of serum nitrate levels in various conditions, such as ischemia, diabetes, and hypertension, has been reported [7,20,21].

Saliva is produced by several specialized glands in the oral cavity and is frequently used to diagnose diseases. It can be collected in a non-invasive manner, thus reducing the stress for both the patient and the operator. Additionally, it may be considered a cost-effective approach for screening large populations [22].

Similar to previous studies [9,22], significant differences in salivary triglyceride, cholesterol, LDL, and HDL levels were observed between hypertensive patients and healthy individuals in the current study. One study, which assessed the lipid peroxidation and antioxidant levels in type 2 diabetes patients, reported significantly higher malondialdehyde levels in the saliva and serum compared to the control group [12]. Kalburgi et al. reported significant differences in salivary cholesterol and triglyceride levels between those with and without periodontitis [8]. Salivary NO is an important regulator of various physiological and pathological mechanisms in the body, and its levels in the oral cavity are known to be associated with several oral diseases [23,24]. In alignment with the findings reported by Nautiyal et al. [25] and Barbadoro et al. [26], a significant (*p* = 0.014) difference in salivary NO levels was observed between hypertensive patients (127.4 ± 4.46 µmol/L) and controls (156.27 ± 5.69 µmol/L) in the current study. Taken together, these findings indicate the potential of saliva as a diagnostic fluid to evaluate various systemic conditions.

Serum triglyceride levels < 150 mg/dL are considered normal but tend to increase with age [27]. A recent review by Spitler et al. reported associations between aging and higher plasma triglyceride levels with decreased clearance in humans and animals [28]. A significant correlation between triglyceride levels and age was observed among the hypertensive patients in the present study; moreover, the levels were significantly different between age groups 40–43 and 47–50 in the same group.

It is worth noting that salivary lipid and NO levels are influenced by various factors, such as age, salivary flow rate, duration of fasting before saliva collection, and method and duration of collection. Serum lipids can reach the saliva via passive diffusion through the capillary wall, interstitial space, basal cell membrane of the acinus cell or duct cell, cytoplasm of the acinus or duct cell, or the luminal cell membrane; alternatively, they can be actively transported via ultrafiltration through intercellular junctions or directly from the gingival crevicular fluid [29]. Moreover, chronic infections like periodontitis can alter the salivary TC, HDL, LDL, and TG levels [8]. In a recent study by Toit et al., dietary intake (green leafy vegetables) significantly altered the oral microbiome and exerted a positive effect on the salivary pH and buffering capacity, proving useful in managing and treating periodontal disease; however, no significant associations between oral microbial changes and blood pressure levels were reported [30]. Recently, periodontitis has been shown to impact the nitrogen reduction capacity of the oral microbiome, thereby affecting the dietary nitrate-derived NO levels in the saliva [31]. These findings indicate that salivary glands and oral bacteria play significant roles in the nitrate–nitrite–NO pathway in the human body [32].

Rahim and Yaacob reported a significant increase in nitrite concentration in saliva under fasting conditions (6 h), which may be attributed to the decrease in salivary flow rate during fasting, impacting the conversion of nitrates to nitrite by the oral bacteria [33,34]. In the present study, saliva samples were collected after about 8 h of fasting. Furthermore, using an antibacterial mouthwash has been shown to inhibit the positive effects of exogenous (dietary) on blood pressure [5]. Another study demonstrated a marked reduction in the conversion of salivary nitrate to nitrite alongside a decrease in circulating plasma nitrite and a significant increase in blood pressure [35], indicating the role of the oral microbiome in cardiovascular health. Interestingly, all patients in the current study had undergone oral prophylaxis at the dental hospital before saliva collection. Thus, the effects of fasting and mouthwash on salivary levels or biomarkers warrant further attention.

One of the limitations of the present study is the absence of comparisons between the serum and the salivary nitrate levels and lipid profiles, which could have strengthened the findings. Nonetheless, correlations between the serum and salivary levels and the role of saliva as a non-invasive diagnostic tool for assessing the lipid profile and nitrate levels have been reported previously [16,29]. A recent study evaluating the use of salivary biomarkers to diagnose cardiovascular disease reported that some biomarkers, such as C-reactive protein, creatine kinase-myocardial band, myoglobin, and troponin I, showed significant promise in diagnosing the condition [36]. Additional studies considering the effect of dietary habits on saliva are warranted.

## 5. Conclusions

This is the first study to quantify the nitrate and lipid profile levels among hypertensive patients in Saudi Arabia. Significant differences were observed between the two groups, indicating that saliva can be considered as a non-invasive diagnostic tool for assessing the nitrate levels and lipid profile in these patients. However, several factors, such as stimulation of saliva, salivary flow variations, use of medications, oral hygiene, duration of fasting before collection, and the method and time of saliva collection, could alter the composition of saliva. Thus, the standardization of saliva collection procedures, storage, and analysis is crucial. Additional multi-center studies with larger sample sizes and more precise testing parameters are required to validate the findings before replacing the salivary diagnostic tests with conventional tests.

## Figures and Tables

**Table 1:** Descriptive statistics of the salivary variables.

Salivary Test Variables		Control			Test		
	N	Minimum	Maximum	Mean ± SD	Minimum	Maximum	Mean ± SD
TRI (mg/dL)	40	50.0	95.0	68.85 ± 12.45	55	180	79.4 ± 20.71
CHO (mg/dL)	40	36	63	52.20 ± 7.19	38	77	55.67 ± 7.14
HDL (mg/dL)	40	9	18	14.38 ± 2.03	9	17	13.93 ± 2.27
LDL (mg/dL)	40	69	99	81.85 ± 8.16	46	116	84.80 ± 12.51
NO (µmol/L)	40	148	165.8	156.27 ± 5.69	122	140.2	127.4 ± 4.46

N, number; SD, standard deviation; TRI, triglycerides; CHO, cholesterol; HDL, high-density lipoproteins; LDL, low-density lipoproteins; NO, nitric oxide.

**Table 2:** Comparisons of the salivary parameters between the hypertensive and control groups.

Salivary Parameters	t	df	*p*-Value
TRI	−2.76	78	0.007 *
CHO	−2.17	78	0.033 *
HDL	0.90	78	0.371
LDL	−1.22	78	0.225
NO	2.47	78	0.014 *

t, independent sample *t*-test; df, degrees of freedom; TRI, triglycerides; CHO, cholesterol; HDL, high-density lipoproteins; LDL, low-density lipoproteins; NO, nitric oxide. * *p* < 0.05.

**Table 3:** Inter-variable Pearson’s correlation test in the test group.

		Age	TRI	CHO	HDL	LDL	NO
Age	Pearson Correlation	1	0.326 *	−0.184	−0.045	−0.131	0.033
	Sig. (2-tailed)		0.040 *	0.255	0.785	0.421	0.840
	N	40	40	40	40	40	40
TRI	Pearson Correlation		1	0.008	−0.059	0.006	−0.179
	Sig. (2-tailed)			0.959	0.716	0.970	0.269
	N		40	40	40	40	40
CHO	Pearson Correlation			1	−0.173	0.155	0.083
	Sig. (2-tailed)				0.285	0.339	0.610
	N			40	40	40	40
HDL	Pearson Correlation				1	−0.153	−0.242
	Sig. (2-tailed)					0.344	0.133
	N				40	40	40
LDL	Pearson Correlation					1	0.196
	Sig. (2-tailed)						0.226
	N					40	40
NO	Pearson Correlation						1
	Sig. (2-tailed)						
	N						40

N, number; TRI, triglycerides; CHO, cholesterol; HDL, high-density lipoproteins; LDL, low-density lipoproteins; NO, nitric oxide. * Correlation is significant at the 0.05 level (2-tailed).

**Table 4:** Comparison of the variables between and among subgroups based on age in the test group.

		Sum of Squares	df	Mean Square	F	Sig.	LSD Post Hoc at 95% CI
TRI	Between subgroups	2802.431	2	1401.215	3.725	0.034 *	1.0–3.0 (*p* = 0.015 *)
	Within subgroups	13,919.169	37	376.194			1.0–2.0 (*p* = 0.652)
	Total	16,721.600	39				
CHO	Between subgroups	61.007	2	30.503	0.596	0.556	-
	Within subgroups	1894.544	37	51.204			
	Total	1955.551	39				
HDL	Between subgroups	0.478	2	0.239	0.043	0.958	-
	Within subgroups	203.470	37	5.499			
	Total	203.948	39				
LDL	Between subgroups	138.188	2	69.094	0.430	0.653	-
	Within subgroups	5939.052	37	160.515			
	Total	6077.240	39				
NO	Between subgroups	41.881	2	20.940	0.842	0.439	-
	Within subgroups	920.270	37	24.872			
	Total	962.151	39				

LSD, least significant difference; df, degrees of freedom; CI, confidence interval; TRI, triglycerides; CHO, cholesterol; HDL, high-density lipoproteins; LDL, low-density lipoproteins; NO, nitric oxide. * Correlation is significant at the 0.05 level (2-tailed).

## Data Availability

The original contributions presented in the study are included in the article, further inquiries can be directed to the corresponding author.
